# Spontaneous Hematomas and Deep Vein Thrombosis during the Recovery from a SARS-CoV-2 Infection: Case Report and Literature Review

**DOI:** 10.3390/medicina58020230

**Published:** 2022-02-02

**Authors:** Cristina Tudoran, Mariana Tudoran, Ahmed Abu-Awwad, Talida Georgiana Cut, Florica Voiță-Mekereș

**Affiliations:** 1 Department VII, Internal Medicine II, University of Medicine and Pharmacy “Victor Babes” Timisoara, E. Murgu Square, Nr. 2, 300041 Timisoara, Romania; tudoran.cristina@umft.ro; 2 Center of Molecular Research in Nephrology and Vascular Disease, Faculty of Medicine, University of Medicine and Pharmacy “Victor Babes” Timisoara, E. Murgu Square, Nr. 2, 300041 Timisoara, Romania; 3 County Emergency Hospital, L. Rebreanu Str., Nr. 156, 300723 Timisoara, Romania; 4 Department XV-Orthopedics Traumatology, Urology and Medical Imaging Internal Medicine II, Faculty of Medicine, University of Medicine and Pharmacy “Victor Babes” Timisoara, E. Murgu Square, Nr. 2, 300041 Timisoara, Romania; ahm.abuawwad@umft.ro; 5 Department XIII, Discipline of Infectious Diseases, University of Medicine and Pharmacy “Victor Babes” Timisoara, E. Murgu Square, Nr. 2, 300041 Timisoara, Romania; 6 Doctoral School, University of Medicine and Pharmacy “Victor Babes” Timisoara, E. Murgu Square, Nr. 2, 300041 Timisoara, Romania; 7 Center for Ethics in Human Genetic Identifications, University of Medicine and Pharmacy “Victor Babes” Timisoara, E. Murgu Square, Nr. 2, 300041 Timisoara, Romania; 8 Morphological Disciplines, Faculty of Medicine and Pharmacy, University of Oradea, 410087 Oradea, Romania; mekeres_florina@yahoo.com

**Keywords:** COVID-19, spontaneous hematoma, deep vein thrombosis, low molecular weight heparins

## Abstract

*Background:* The frequent occurrence of thromboembolic events in patients infected with the severe acute respiratory syndrome CoV2 (SARS-CoV-2) virus is a well-recognized fact in the medical literature, but less data is available about possible hemorrhagic incidents. *Methods:* We report the case of a 76-year-old patient who suffered from a mild COVID-19 infection in September 2021 and after four weeks, experienced a completely spontaneous popliteal hematoma followed by deep vein thrombosis (DVT). Therapy with low molecular weight heparins (LMWH) was started, but subsequently, the patient developed a massive sub-pectoral and calf hematoma leading to moderate post-hemorrhagic anemia and acute kidney injury. This patient was treated completely conservatively. *Conclusions:* Considering the continuous spread of the infection with various, continuously evolving strains of this virus and the extended use of LWMH in clinical practice, such cases were seldom described in the medical literature, but should be considered as a potential cause for hemorrhagic events.

## 1. Introduction

It is a general consensus that the infection with the severe acute respiratory syndrome CoV-2 (SARS-CoV-2) virus is associated with an increased risk for thromboembolic events such as deep vein thrombosis (DVT) or even pulmonary embolism (PE), numerous scientific researches being published about this topic [1,2,3,4]. Responsible mechanisms are considered: (a) an increased release of the vasoconstrictor angiotensin II, mediated by the virus, (b) a decreased level of angiotensin which acts as a vasodilator, (c) the sepsis-induced release of cytokines that can even result in an exuberant “cytokine storm”, and (d) inflammatory responses that may trigger a coagulopathy in COVID-19. Hypercoagulability is an important hallmark of inflammation. Pro-inflammatory cytokines are critically involved in abnormal clot formation and platelet hyperactivation and also play an important role in the downregulation of important physiological anticoagulant mechanisms. It has been determined that pro-inflammatory cytokines such as interleukin 6 (IL-6), IL-17A, and tumor necrosis factor reached increased levels in the majority of patients with severe outcomes [2,3,5].

According to guidelines recommendations, anticoagulant drugs, especially low molecular weight heparins (LMWH), in prophylactic or even therapeutic doses, should be started as soon as possible after confirming the SARS-CoV-2 infection to prevent the occurrence of life-threatening complication [6,7,8]. Nevertheless, data over hemostasis disturbances related to COVID-19 are scarce in the medical literature [3,9,10,11]. Except for the severe examples of disseminated intravascular coagulopathy observed in severe cases hospitalized in the intensive care units, further hemorrhages with diverse locations have been reported [12,13]. In the same vein, spontaneous hematomas developed in patients suffering or recovering from COVID-19 were rarely described [7,14], being mostly associated with the treatment with LMWH [1,9,11]. According to the guidelines, LMWH are the treatment of choice for patients with deep vein thrombosis (DVT), and are also used as prophylaxis in COVID-19 [15,16,17]. This therapy should be initiated as soon as possible after establishing the diagnosis to prevent DVT and life-threatening complications, especially pulmonary embolisms (PEs) [1,3,18]. Unfortunately, this therapy used to treat the coagulopathy related to endothelial injury and inflammation may increase the risk of bleeding in elderly patients. Advanced age is often associated with a prothrombotic state, increased fibrinolytic activity in friable, prone to rupture atherosclerotic blood vessels. Other contributing factors are impaired renal function and some concomitant medication (anticoagulants, antiplatelet therapy and calcium channel blockers).

## 2. Case Presentation

**Figure 1:**
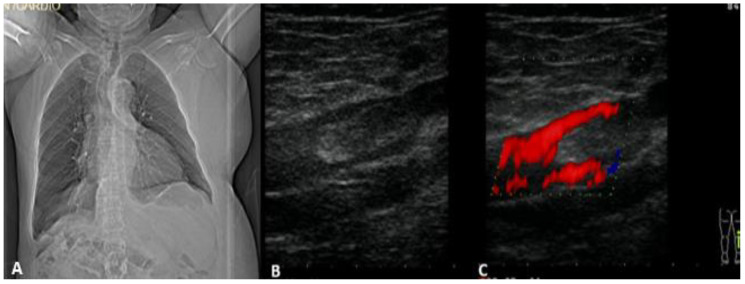
(**A**) Chest computed-tomography evidencing abdominal situs inversus; (**B**) Immage of a thrombus in the vein; (**C**) venous Doppler exam evidencing a thrombus in thevein.

A 76-year-old patient in good health condition, who suffered in September 2021 a mild form of SARS-CoV-2 infection, for which he received prophylactic therapy with Eliquis 2.5 mg twice daily to prevent thromboembolic events, but stopped this treatment after two weeks by his own decision, developed a spontaneous left popliteal hematoma when hurrying to cross the street. The patient was already diagnosed with abdominal situs inversus (Figure 1, left) and systemic hypertension grade II, and had surgery for a liver hydatid cyst 20 years ago.

**Figure 2:**
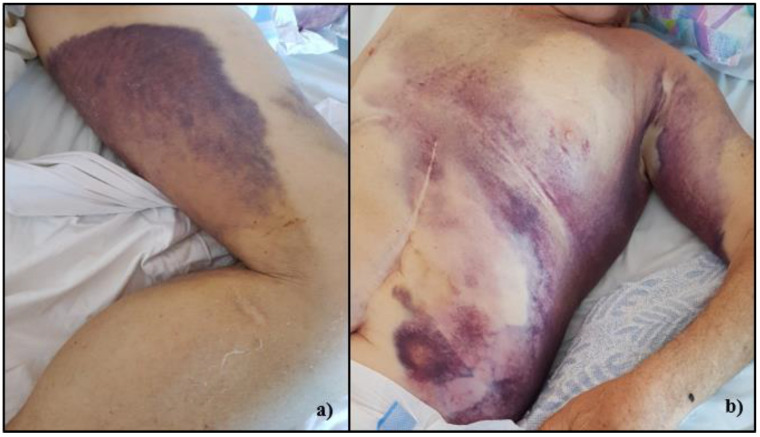
(**a**) left popliteal hematoma with suffusions in the thigh and deep vein thrombosis of the left calf; (**b**) massive left sub-pectoral hematoma.

Conservative measures were recommended, but after three days he developed an increasing pain and swelling in the left calf, severely limiting walking. He went to the emergency room and was diagnosed with DVT of the left popliteal vein, confirmed by vascular sonography, (Figure 1, right). He was prescribed therapy with subcutaneous Enoxaparin, 80 mg twice daily. After the second day of therapy the pain and tumefaction of the calf diminished. In the seventh day of treatment, the patient complained about chest pain, associated with new onset of the left calf pain, and came to the emergency room. At the clinical exam, at the left pectoral region and left calf bruising were observed, and pain and tumefaction were palpable (Figure 2a,b). On the electrocardiogram, there was no evidence of ischemic changes, and the markers of myocardial injury were negative.

**Figure 3:**
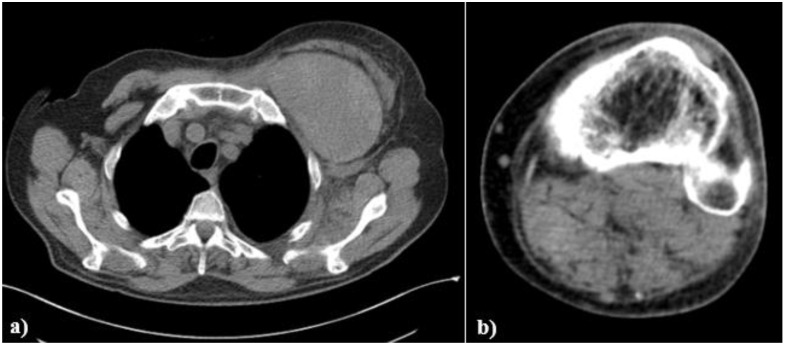
(**a**) Thorax CT scan evidencing left sub-pectoral hematoma; (**b**) computed tomography of the left calf showing a profound hematoma.

The chest computed-tomography (CT) showed a massive sub-pectoral hematoma, with compression of the surrounding tissue (Figure 3a), and the CT of the left calf confirmed the presence of a new non-compressive hematoma (Figure 3b).

A second venous ultrasound revealed the dissolution of the popliteal thrombus but showed a profound calf hematoma. Blood tests revealed anemia with a decline of the hemoglobin level from 12.8 to 9.3 mg/dL, associated with worsening of renal function (creatinine 2.6 mg/dL) due to hemolysis and rhabdomyolysis. Hospitalization was proposed, but the patient refused it.

Subcutaneous LMWH and anti-hypertensive drugs were stopped, oral hydration and antibiotics were recommended together with local therapeutic measures with heparin gel and ice applications. After three days, pain and swelling diminished gradually, and the hematomas reduced in size with good clinical and biological resolution.

Follow-up and outcomes. After a week, the symptoms and hematomas diminished without need of surgery and renal function improved. It was decided to further withhold anticoagulant treatment and the patient was treated conservatively.

## 3. Discussion

The increased prevalence of thromboembolic events in patients infected with the SARS-CoV-2 virus was observed since the early stages of the COVID-19 and was debated in numerous studies [5,19,20]. As early as the spring of 2020, several extensive papers, such as the one of Miesbach et al. [5], analyzed early reports [2,11,21] and discussed over a prevalence ranging largely, from 17.7%, to 31% or even 40% of patients with pneumonia due to the SARS-CoV-2 infection. Increased levels of D-Dimers were observed in even more patients, approximately 49% [5]. It is a general consensus that anticoagulant therapy, mostly LMWH and direct factor Xa inhibitors, should be recommended for the prophylaxis of thromboembolic events in patients with symptomatic forms of COVID-19 [1,3,11]. According to guidelines recommendations [1], extended prophylaxis with LMWH or direct oral anticoagulants is prescribed in COVID-19 patients to reduce the risk of DVT and PE after the discharge from the hospital with the risk of increasing bleeding events, including major bleeding. Although no specific data for the use of anticoagulants in patients with mild/moderate forms of COVID-19 exist, it is reasonable to recommend an extended prophylaxis according to the individualized thrombotic/hemorrhagic risk [15,19]. It is still discussed that these doses do not always seem effective, and DVP and/or PE were described even during recovery [20,22,23]. In contrast, the topic of hemostasis disturbances and increased risk of bleeding related to COVID-19 is less discussed in the medical literature. In critically ill patients, a higher incidence of disseminated intravascular coagulopathy, and/hemorrhagic complications have been reported [8,10,24,25]. Even rare cases with subcutaneous hematomas developed during the acute phase of the disease, related mostly to the administration of anticoagulants have been described [11,17,26,27,28]. While thrombotic events occurred early, in the first week of the acute illness, it was observed that hematomas appeared after 2–3 weeks, mostly in elderly people with comorbidities [27,28].

The case presented in this paper is peculiar because the patient developed a completely spontaneous popliteal hematoma a month after he tested positive for a SARS-CoV-2 infection, and experienced a mild form of this disease without pneumonia. It is still to be discussed that he was treated with prophylactic doses of apixaban during the acute illness, but had interrupted this therapy two weeks prior to this event. It is probable that advanced age and associated systemic hypertension promoting endothelial dysfunction were contributing factors for the occurrence of the hematoma. Afterwards, at the same limb, DVT was diagnosed, and therapy with LMWH was started as recommended in the guidelines [18]. It is possible that the occurrence of DVT was favored by the compression of the popliteal vein exerted by the hematoma and by its immobilization. 

Unexpectedly, a few days after starting therapy with LMWH, he developed massive hematomas located in the pectoral region, and left thigh and calf. The occurrence of spontaneous hematomas after therapy with LMWH is an equally rare medical condition, these complications developing usually at the injection site. Few cases of massive, spontaneous hematomas have been reported in patients receiving LMWH—they occur mostly in the abdominal region [29,30,31] and in frail, elderly patients (over 80 years old). It is very likely that the recent infection with the SARS-CoV-2 virus, with its related thrombosis and hemostasis disturbances, could be responsible for this succession of events. We found no other case reports in the literature with a completely spontaneous popliteal hematoma followed by DVT, who subsequently developed a new massive sub-pectoral and calf hematomas leading to anemia and acute kidney injury during the recovery from COVID-19. Fortunately, our patient had a favorable evolution under conservative therapy with no need of further surgery. Certainly, there is no indisputable proof that the occurrence of these hematomas was determined only by COVID-19, but their development during the recovery strongly suggests an important contribution of this disease.

## 4. Conclusions

Thromboembolic events are frequently encountered in patients infected with the SARS-CoV-2 virus, occurring mostly in the acute illness, and seldom during the recovery. Our case represents an unusual succession of spontaneous hematomas and DVP determining anemia and acute kidney injury during recovery from COVID-19. 

## Data Availability

Not applicable.
